# The *Global Alliance for Infections in Surgery:* defining a model for antimicrobial stewardship—results from an international cross-sectional survey

**DOI:** 10.1186/s13017-017-0145-2

**Published:** 2017-08-01

**Authors:** Massimo Sartelli, Francesco M. Labricciosa, Pamela Barbadoro, Leonardo Pagani, Luca Ansaloni, Adrian J. Brink, Jean Carlet, Ashish Khanna, Alain Chichom-Mefire, Federico Coccolini, Salomone Di Saverio, Addison K. May, Pierluigi Viale, Richard R. Watkins, Luigia Scudeller, Lilian M. Abbo, Fikri M. Abu-Zidan, Abdulrashid K. Adesunkanmi, Sara Al-Dahir, Majdi N. Al-Hasan, Halil Alis, Carlos Alves, André R. Araujo da Silva, Goran Augustin, Miklosh Bala, Philip S. Barie, Marcelo A. Beltrán, Aneel Bhangu, Belefquih Bouchra, Stephen M. Brecher, Miguel A. Caínzos, Adrian Camacho-Ortiz, Marco Catani, Sujith J. Chandy, Asri Che Jusoh, Jill R. Cherry-Bukowiec, Osvaldo Chiara, Elif Colak, Oliver A. Cornely, Yunfeng Cui, Zaza Demetrashvili, Belinda De Simone, Jan J. De Waele, Sameer Dhingra, Francesco Di Marzo, Agron Dogjani, Gereltuya Dorj, Laurent Dortet, Therese M. Duane, Mutasim M. Elmangory, Mushira A. Enani, Paula Ferrada, J. Esteban Foianini, Mahir Gachabayov, Chinmay Gandhi, Wagih Mommtaz Ghnnam, Helen Giamarellou, Georgios Gkiokas, Harumi Gomi, Tatjana Goranovic, Ewen A. Griffiths, Rosio I. Guerra Gronerth, Julio C. Haidamus Monteiro, Timothy C. Hardcastle, Andreas Hecker, Adrien M. Hodonou, Orestis Ioannidis, Arda Isik, Katia A. Iskandar, Hossein S. Kafil, Souha S. Kanj, Lewis J. Kaplan, Garima Kapoor, Aleksandar R. Karamarkovic, Jakub Kenig, Ivan Kerschaever, Faryal Khamis, Vladimir Khokha, Ronald Kiguba, Hong B. Kim, Wen-Chien Ko, Kaoru Koike, Iryna Kozlovska, Anand Kumar, Leonel Lagunes, Rifat Latifi, Jae G. Lee, Young R. Lee, Ari Leppäniemi, Yousheng Li, Stephen Y. Liang, Warren Lowman, Gustavo M. Machain, Marc Maegele, Piotr Major, Sydney Malama, Ramiro Manzano-Nunez, Athanasios Marinis, Isidro Martinez Casas, Sanjay Marwah, Emilio Maseda, Michael E. McFarlane, Ziad Memish, Dominik Mertz, Cristian Mesina, Shyam K. Mishra, Ernest E. Moore, Akutu Munyika, Eleftherios Mylonakis, Lena Napolitano, Ionut Negoi, Milica D. Nestorovic, David P. Nicolau, Abdelkarim H. Omari, Carlos A. Ordonez, José-Artur Paiva, Narayan D. Pant, Jose G. Parreira, Michal Pędziwiatr, Bruno M. Pereira, Alfredo Ponce-de-Leon, Garyphallia Poulakou, Jacobus Preller, Céline Pulcini, Guntars Pupelis, Martha Quiodettis, Timothy M. Rawson, Tarcisio Reis, Miran Rems, Sandro Rizoli, Jason Roberts, Nuno Rocha Pereira, Jesús Rodríguez-Baño, Boris Sakakushev, James Sanders, Natalia Santos, Norio Sato, Robert G. Sawyer, Sandro Scarpelini, Loredana Scoccia, Nusrat Shafiq, Vishalkumar Shelat, Costi D. Sifri, Boonying Siribumrungwong, Kjetil Søreide, Rodolfo Soto, Hamilton P. de Souza, Peep Talving, Ngo Tat Trung, Jeffrey M. Tessier, Mario Tumbarello, Jan Ulrych, Selman Uranues, Harry Van Goor, Andras Vereczkei, Florian Wagenlehner, Yonghong Xiao, Kuo-Ching Yuan, Agnes Wechsler-Fördös, Jean-Ralph Zahar, Tanya L. Zakrison, Brian Zuckerbraun, Wietse P. Zuidema, Fausto Catena

**Affiliations:** 1 Department of Surgery, Macerata Hospital, Macerata, Italy; 2 Department of Biomedical Sciences and Public Health, Unit of Hygiene, Preventive Medicine and Public Health, Università Politecnica delle Marche, Ancona, Italy; 3 Infectious Diseases Unit, Bolzano Central Hospital, Bolzano, Italy; 4 General Surgery Department, Papa Giovanni XXIII Hospital, Bergamo, Italy; 5 Department of Clinical microbiology, Ampath National Laboratory Services, Milpark Hospital, Johannesburg, South Africa; 6 Division of Infectious Diseases and HIV Medicine, Department of Medicine, University of Cape Town, Cape town, South Africa; 7 World Alliance against Antibiotics Resistance, Rome, Italy; 8 Center for Critical Care, Anaesthesiology Institute and Department of Outcomes Research, Cleveland Clinic, Cleveland, OH USA; 9 Department of Surgery and Obstetrics/Gynaecology, Regional Hospital, Limbe, Cameroon; 10 Department of Surgery, Infermi Hospital, Rimini, Italy; 11 Department of Surgery, Maggiore Hospital, Bologna, Italy; 12 Department of Surgery, Vanderbilt University Medical Center, Nashville, Tennessee USA; 13 Infectious Diseases Unit, Department of Medical and Surgical Sciences, Sant’Orsola Hospital, University of Bologna, Bologna, Italy; 14 Division of Infectious Diseases, Cleveland Clinic Akron General, Akron, OH USA; 15 Department of Medicine, Northeast Ohio Medical University, Rootstown, OH USA; 16 Clinical Epidemiology Unit, IRCCS Policlinico San Matteo Foundation, Pavia, Italy; 17 Division of Infectious Diseases, Jackson Health System, University of Miami Miller School of Medicine, Miami, FL USA; 18 Department of Surgery, College of Medicine and Health Sciences, UAE University, Al-Ain, United Arab Emirates; 19 Department of Surgery, College of Health Sciences, Obafemi Awolowo University, Ile-Ife, Nigeria; 20 Division of Clinical and Administrative Sciences, College of Pharmacy, Xavier University of Louisiana, New Orleans, LA USA; 21 Department of Medicine, Division of Infectious Diseases, University of South Carolina School of Medicine, Columbia, SC USA; 22 General Surgery Department, Bakirkoy Dr Sadi Konuk Training and Research Hospital, Instanbul, Turkey; 23 Unit of Prevention and Infection Control, Center of Hospital Epidemiology, São João Hospital Centre, Porto, Portugal; 24 Infection Control Committee, Prontobaby Hospital da Criança, Rio de Janeiro, Brazil; 25 Department of Surgery, University Hospital Center, Zagreb, Croatia; 26 Trauma and Acute Care Surgery Unit, Hadassah Hebrew University Medical Center, Jerusalem, Israel; 27 Department of Surgery, Weill Cornell Medicine, New York, NY USA; 28 Department of General Surgery, Hospital San Juan de Dios de La Serena, La Serena, Chile; 29 Academic Department of Surgery, Queen Elizabeth Hospital, Birmingham, UK; 30 Department of Microbiology National Reference Laboratory Cheikh Khalifa Ibn Zaid Hospital, Mohammed 6th University of Health Sciences, Casablanca, Morocco; 31 Department of Pathology and Laboratory Medicine, VA Boston HealthCare System, Boston, MA USA; 32 Department of Pathology and Laboratory Medicine, Boston University School of Medicine, Boston, MA USA; 33 Department of Surgery, Hospital Clínico Universitario, Santiago de Compostela, Spain; 34 Hospital Epidemiology and Infectious Diseases, Hospital Universitario Dr Jose Eleuterio Gonzalez, Monterrey, Mexico; 35 Department of Emergency, Umberto I Hospital, Rome, Italy; 36 Department of Pharmacology, Pushpagiri Institute of Medical Sciences and Research Centre, Thiruvalla, Kerala India; 37 Department of General Surgery, Kuala Krai Hospital, Kuala Krai, Kelantan Malaysia; 38 Division of Acute Care Surgery, Department of Surgery, University of Michigan, Ann Arbor, MI USA; 39 Niguarda Hospital, Milano, Italy; 40 Department of General Surgery, Health Sciences University, Samsun Training and Research Hospital, Samsun, Turkey; 41 Department of Internal Medicine and Infectious Diseases, University of Cologne, Cologne, Germany; 42 Department of Surgery, Tianjin Nankai Hospital, Nankai Clinical School of Medicine, Tianjin Medical University, Tianjin, China; 43 Department General Surgery, Kipshidze Central University Hospital, Tbilisi, Georgia; 44 Department of Digestive Surgery, Cannes Hospital, Cannes, France; 45 Department of Critical Care Medicine, Ghent University Hospital, Ghent, Belgium; 46 School of Pharmacy, Faculty of Medical Sciences, The University of the West Indies, St. Augustine, Trinidad and Tobago; 47 Eric Williams Medical Sciences Complex, Uriah Butler Highway, Champ Fleurs, Trinidad and Tobago; 48 Department of Surgery, Versilia Hospita, Lido di Camaiore, Italy; 49 Department of Surgery, University Hospital of Trauma, Tirana, Albania; 50 School of Pharmacy and Biomedicine, Mongolian National University of Medical Sciences, Ulaanbaatar, Mongolia; 51 Department of Microbiology, Bicêtre Hospital, Paris-Sud University, La Kremlin-Bicêtre, France; 52 Department of Surgery, John Peter Smith Health Network, Fort Worth, Texas USA; 53 Sudan National Public Health Laboratory, Federal Ministry of Health, Khartoum, Sudan; 54 Department of Medicine, Infectious Disease Division, King Fahad Medical City, Riyadh, Saudi Arabia; 55 Department of Surgery, Virginia Commonwealth University, Richmond, VA USA; 56 Department of Surgery, Clinica Foianini, Santa Cruz, Bolivia; 57 Department of Abdominal Surgery, Vladimir City Clinical Hospital of Emergency Medicine, Vladimir, Russia; 58 Department of Surgery, Bharati Vidyapeeth Deemed University Medical College and Hospital, Sangli, Maharashtra India; 59 Department of General Surgery, Mansoura Faculty of Medicine, Mansoura University, Mansoura, Egypt; 60 Sixth Department of Internal Medicine, Hygeia General Hospital, Athens, Greece; 61 Second Department of Surgery, Aretaieion University Hospital, National and Kapodistrian University of Athens, Athens, Greece; 62 Center for Global Health, Mito Kyodo General Hospital, University of Tsukuba, Mito, Ibaraki Japan; 63 University Department for Tumours, Sestre Milosrrdnice UHC, Zagreb, Croatia; 64 General and Upper GI Surgery, Queen Elizabeth Hospital, Birmingham, UK; 65 Peruvian Navy Medical Center, Lima, Peru; 66 Department of Gastrointestinal Surgery, Santa Casa Hospital, Campo Grande, Brazil; 67 Trauma and Trauma ICU, Inkosi Albert Luthuli Central Hospital and Department of Surgery, University of KwaZulu-Natal, Durban, South Africa; 68 Department of General and Thoracic Surgery, University Hospital Giessen, Giessen, Germany; 69 Department of Surgery, Faculty of Medicine, University of Parakou, BP 123 Parakou, Benin; 70 Fourth Surgical Department, General Hospital G. Papanikolaou, Medical School, Aristotle University of Thessaloniki, Thessaloniki, Greece; 71 Department of General Surgery, Erzincan University, Faculty of Medicine, Erzincan, Turkey; 72 Department of Pharmacy, Lebanese, International University, Beirut, Lebanon; 73 Drug Applied Research Center, Tabriz University of Medical Sciences, Tabriz, Iran; 74 Division of Infectious Diseases, American University of Beirut, Beirut, Lebanon; 75 Department of Surgery Philadelphia VA Medical Center, Perelman School of Medicine, University of Pennsylvania, Philadelphia, PA USA; 76 Department of Microbiology, Gandhi Medical College, Bhopal, India; 77 Clinic for Emergency Surgery, Medical Faculty University of Belgrade, Belgrade, Serbia; 78 Third Department of General Surgery, Jagiellonian University Medical College, Krakow, Poland; 79 Department of Abdominal Surgery, Regional Hospital of Tienen, Tienen, Belgium; 80 Department of Internal Medicine, Royal Hospital, Muscat, Oman; 81 Department of Emergency Surgery, City Hospital, Mozyr, Belarus; 82 Department of Pharmacology and Therapeutics, College of Health Sciences, Makerere University, Kampala, Uganda; 83 Department of Internal Medicine, Seoul National University Bundang Hospital, Seongnam, Republic of Korea; 84 Department of Internal Medicine, National Cheng Kung University Hospital, Tainan, Taiwan; 85 Department of Primary Care and Emergency Medicine, Kyoto University Graduate School of Medicine, Kyoto, Japan; 86 Department of Surgery n. 2, Higher educational institutions of Ukraine Bukovina State Medical University, Chernivtci City, Ukraine; 87 Section of Critical Care Medicine and Section of Infectious Diseases, Department of Medicine, Medical Microbiology and Pharmacology/Therapeutics, University of Manitoba, Winnipeg, MB Canada; 88 Hospital Central Dr Ignacio Morones Prieto, San Luis Potosi, Mexico; 89 Department of Surgery, Division of Trauma, University of Arizona, Tucson, AZ USA; 90 Department of Surgery, Yonsei University College of Medicine, Seoul, South Korea; 91 Texas Tech University, Health Sciences Center School of Pharmacy, Abilene, TX USA; 92 Abdominal Center, University Hospital Meilahti, Helsinki, Finland; 93 Department of Surgery, Inling Hospital, Nanjing University School of Medicine, Nanjing, China; 94 Division of Infectious Diseases, Division of Emergency Medicine, Washington University School of Medicine, St. Louis, MO USA; 95 Clinical Microbiology and Infectious Diseases, School of Pathology, Faculty of Health Sciences, University of the Witwatersrand, Johannesburg, South Africa; 96 Department of Surgery, Universidad Nacional de Asuncion, Asuncion, Paraguay; 97 Department for Traumatology and Orthopedic Surgery, Cologne Merheim Medical Center (CMMC), University of Witten/Herdecke (UW/H), Cologne, Germany; 98 Second Department of General Surgery, Jagiellonian University Medical College, Krakow, Poland; 99 Health Research Program, Institute of Economic and Social Research, University of Zambia, Lusaka, Zambia; 100 Clinical Research Center, Fundacion Valle del Lili, Cali, Colombia; 101 First Department of Surgery, Tzaneion General Hospital, Piraeus, Greece; 102 Service of General Surgery, Hospital Complex of Jaén, Jaén, Spain; 103 Department of Surgery, Post-Graduate Institute of Medical Sciences, Rohtak, India; 104 Servicio de Anestesia y Reanimación, Hospital Universitario La Paz Madrid, Madrid, Spain; 105 Department of Surgery, Radiology, University Hospital of the West Indies, Kingston, Jamaica; 106 Infectious Diseases Division, Department of Medicine, Prince Mohamed Bin Abdulaziz Hospital, Ministry of Health, Riyadh, Saudi Arabia; 107 Departments of Medicine, Clinical Epidemiology and Biostatistics, and Pathology and Molecular Medicine, McMaster University, Hamilton, ON Canada; 108 Second Surgical Clinic, Emergency Hospital of Craiova, Craiova, Romania; 109 Department of Microbiology, Tribhuvan University Teaching Hospital, Institute of Medicine, Kathmandu, Nepal; 110 Department of Surgery, University of Colorado, Denver Health Medical Center, Denver, CO USA; 111 Department of Surgery, Onandjokwe Hospital, Ondangwa, Namibia; 112 Infectious Diseases Division, Warren Alpert Medical School of Brown University, Rhode Island Hospital, Providence, RI USA; 113 Department of Surgery, University of Michigan, Ann Arbor, MI USA; 114 Department of Surgery, Emergency Hospital of Bucharest, Bucharest, Romania; 115 Clinic for General Surgery, Clinical Centre, Nis, Serbia; 116 Center of Anti-Infective Research and Development, Hartford, CT USA; 117 Department of Surgery, King Abdullah University Hospital, Irbid, Jordan; 118 Department of Surgery and Critical Care, Universidad del Valle, Fundación Valle del Lili, Cali, Colombia; 119 Intensive Care Medicine Department, Centro Hospitalar São João, University of Porto, Porto, Portugal; 120 Department of Microbiology, Grande International Hospital, Dhapasi, Kathmandu, Nepal; 121 Department of Surgery, Santa Casa de Sao Paulo School of Medical Sciences, São Paulo, Brazil; 122 Department of General and Emergency Surgery, University Hospital Kraków, Kraków, Poland; 123 Department of Surgery, University of Campinas, Campinas, Brazil; 124 Laboratory of Clinical Microbiology, Department of Infectious Diseases, Instituto Nacional de Ciencias Médicas y Nutrición Salvador Zubirán, Mexico City, Mexico; 125 Fourth Department of Internal Medicine and Infectious Diseases Unit, National and Kapodstrian University-Medical School, Attikon University General Hospital, Athens, Greece; 126 John Farman Intensive Care Unit, University Hospitals, NHS Foundation Trust, Cambridge, UK; 127 Infectious and Tropical Diseases Department, University Hospital of Nancy, and EA 4360 APEMAC, Lorraine University, Nancy, France; 128 Department of General and Emergency Surgery, Riga East University Hospital ‘Gailezers’, Riga, Latvia; 129 Department of Trauma, Hospital Santo Tomas, Panama, Panama; 130 National Institute for Health Research, Health Protection Research Unit in Healthcare Associated Infections and Antimicrobial Resistance, Imperial College London, Hammersmith Campus, London, UK; 131 Emergency Post-operative Department, Otavio de Freitas Hospital and Hosvaldo Cruz Hospital, Recife, Brazil; 132 Department of General Surgery, Jesenice General Hospital, Jesenice, Slovenia; 133 Trauma and Acute Care Service, St Michael’s Hospital, University of Toronto, Toronto, Canada; 134 Burns, Trauma and Critical Care Research Centre, The University of Queensland, Brisbane, Queensland Australia; 135 Unidad Clínica Intercentros de Enfermedades Infecciosas, Microbiología y Medicina Preventiva, Hospitales Universitarios Virgen Macarena y Virgen del Rocío-IBiS and Departamento de Medicina, Universidad de Sevilla, Seville, Spain; 136 General Surgery Department, Medical University, University Hospital St George, Plovdiv, Bulgaria; 137 JPS Health Network, Texas, TX USA; 138 Hospital Center Tondela Viseu, Tondela, Portugal; 139 Department of Aeromedical Services for Emergency and Trauma Care, Ehime University Graduate School of Medicine, Ehime, Japan; 140 Department of Surgery, University of Virginia Health System, Charlottesville, VA USA; 141 Department of Surgery, University of Sao Paulo, Ribeirao Preto, Brazil; 142 Unit of Hospital Pharmacy, Macerata Hospital, Macerata, Italy; 143 Department of Pharmacology, Postgraduate Institute of Medical Education and Research, Chandigarh, India; 144 Department of General Surgery, Tan Tock Seng Hospital, Tan Tock Seng, Singapore; 145 Office of Hospital Epidemiology/Infection Prevention and Control, University of Virginia Health System, Charlottesville, VA USA; 146 Department of Surgery, Faculty of Medicine, Thammasat University Hospital, Thammasat University, Pathum Thani, Thailand; 147 Department of Gastrointestinal Surgery, Stavanger University Hospital, Stavanger, Norway; 148 Department of Clinical Medicine, University of Bergen, Bergen, Norway; 149 Department of Emergency Surgery and Critical Care, Centro Medico Imbanaco, Cali, Colombia; 150 Department of Surgery, School of Medicine, Pontifícia Universidade Católica do Rio Grande do Sul (PUCRS), Porto Alegre, Brazil; 151 Department of Surgery, North Estonia Medical Center, Tallinn, Estonia; 152 Department of Molecular Biology, Tran Hung Dao Hospital, No 1, Tran Hung Dao Street, Hai Ba Trung Dist, Hanoi, Vietnam; 153 Department of Infectious Diseases, John Peter Smith Health Network, Fort Worth, Texas USA; 154 Institute of Infectious Diseases, Catholic University, Rome, Italy; 155 First Department of Surgery—Department of Abdominal, Thoracic Surgery and Traumatology, General University Hospital, Prague, Czech Republic; 156 Department of Surgery, Medical University of Graz, Graz, Austria; 157 Department of Surgery, Radboud University Nijmegen Medical Center, Nijmegen, The Netherlands; 158 Department of Surgery, Medical School University of Pécs, Pécs, Hungary; 159 Department of Urology, Pediatric Urology and Andrology, Medical Faculty of the Justus Liebig University Giessen, Giessen, Germany; 160 State Key Laboratory for Diagnosis and Treatment of Infectious Diseases, The First Affilliated Hospital, Zhejiang University, Zhejiang, China; 161 Trauma and Emergency Surgery Department, Chang Gung Memorial Hospital, Taoyuan City, Taiwan; 162 Department of Antibiotics and Infection Control, Rudolfstiftung Hospital, Vienna, Austria; 163 Infection Control Unit, Angers University, CHU d’Angers, Angers, France; 164 Division of Trauma and Surgical Critical Care, DeWitt Daughtry Family Department of Surgery, University of Miami, Miami, FL USA; 165 Department of Surgery, University of Pittsburgh, Pittsburgh, PA USA; 166 VU University Medical Center, Amsterdam, The Netherlands; 167 Department of General Surgery, Maggiore Hospital, Parma, Italy

**Keywords:** Antibiotics, Infections, Surgery, Antimicrobial stewardship

## Abstract

**Background:**

Antimicrobial Stewardship Programs (ASPs) have been promoted to optimize antimicrobial usage and patient outcomes, and to reduce the emergence of antimicrobial-resistant organisms. However, the best strategies for an ASP are not definitively established and are likely to vary based on local culture, policy, and routine clinical practice, and probably limited resources in middle-income countries. The aim of this study is to evaluate structures and resources of antimicrobial stewardship teams (ASTs) in surgical departments from different regions of the world.

**Methods:**

A cross-sectional web-based survey was conducted in 2016 on 173 physicians who participated in the AGORA (Antimicrobials: A Global Alliance for Optimizing their Rational Use in Intra-Abdominal Infections) project and on 658 international experts in the fields of ASPs, infection control, and infections in surgery.

**Results:**

The response rate was 19.4%. One hundred fifty-six (98.7%) participants stated their hospital had a multidisciplinary AST. The median number of physicians working inside the team was five [interquartile range 4–6]. An infectious disease specialist, a microbiologist and an infection control specialist were, respectively, present in 80.1, 76.3, and 67.9% of the ASTs. A surgeon was a component in 59.0% of cases and was significantly more likely to be present in university hospitals (89.5%, *p* < 0.05) compared to community teaching (83.3%) and community hospitals (66.7%). Protocols for pre-operative prophylaxis and for antimicrobial treatment of surgical infections were respectively implemented in 96.2 and 82.3% of the hospitals. The majority of the surgical departments implemented both persuasive and restrictive interventions (72.8%). The most common types of interventions in surgical departments were dissemination of educational materials (62.5%), expert approval (61.0%), audit and feedback (55.1%), educational outreach (53.7%), and compulsory order forms (51.5%).

**Conclusion:**

The survey showed a heterogeneous organization of ASPs worldwide, demonstrating the necessity of a multidisciplinary and collaborative approach in the battle against antimicrobial resistance in surgical infections, and the importance of educational efforts towards this goal.

**Electronic supplementary material:**

The online version of this article (doi:10.1186/s13017-017-0145-2) contains supplementary material, which is available to authorized users.

## Background

Antimicrobial Stewardship Programs (ASPs) have been promoted to optimize antimicrobial usage and patient outcomes and reduce the emergence of antimicrobial-resistant organisms. However, the best strategies for an ASP are not definitively established and are likely to vary based on local culture, policy and routine clinical practice, and probably limited resources in middle-income countries [1, 2]. Many hospitals remain without formal programs and those that do continue to struggle with gaining acceptance across service lines [3]. Moreover, identifying optimal efforts to impact system change has been challenging [4].

Restriction strategies may be effective at controlling use but raise issues of prescriber autonomy and require a large personnel commitment. Encouraging multidisciplinary collaboration within health systems to ensure that prophylactic, empirical, and targeted use of antimicrobial agents results in optimal patient outcomes is mandatory in the current era of antimicrobial resistance.

A panel of experts from the Surgical Infection Society (SIS) and World Society of Emergency Surgery (WSES) has recently published a review with the aim of defining the role of surgeons within the ASPs. The panel proposed that the best means of improving antimicrobial stewardship in surgical units worldwide should involve collaboration among various specialties within institutions including prescribing clinicians and pharmacists [5].

In 2016, a multidisciplinary task force from 79 different countries joined a global project to develop a consensus on the rational use of antimicrobials for patients with intra-abdominal infections (IAIs). The project has been termed AGORA (Antimicrobials: A Global Alliance for Optimizing their Rational Use in Intra-Abdominal Infections) [1].

Recently the *Global Alliance for Infections in Surgery* was founded and experts from 87 countries worldwide joined the highly diverse and skilled International Advisory Board. This alliance, promoted by the WSES, includes an interdisciplinary group of hospital administrators, epidemiologists, infection control specialists, infectious disease specialists, microbiologists, clinical pharmacologists and hospital pharmacists, surgeons, and intensivists. The mission of this alliance is to educate healthcare providers promoting the standards of care in managing infections in surgery worldwide [6]. Therefore, this study was conducted to evaluate the structure and resources of antimicrobial stewardship teams (ASTs) in surgical departments from different regions of the world.

## Methods

We conducted a cross-sectional electronic survey evaluating the structure and resources of ASTs in surgical departments. The survey was designed by a multidisciplinary team of investigators including an epidemiologist, a surgeon, an infectious diseases physician, a pharmacologist, and a microbiologist. The questionnaire was piloted among five physicians for face and content validity.

The 24-item self-administered questionnaire collected information from multidisciplinary experts—mostly physicians—about characteristics and composition of the hospital team, implementation of local procedures, availability of antimicrobial use monitoring and surveillance systems, presence of an ASP, and related interventions (Additional file 1). An electronic invitation with a link to the survey was sent to 831 physicians: 173 physicians who participated in the AGORA project [1], and a large number (658) of international experts in the fields of antimicrobial stewardship, infection control, and infections in surgery identified after a thorough investigation using the PubMed database. The survey was Internet-based (using http://www.docs.google.com). Participation was voluntary but not anonymous; however, the confidentiality of respondents and their choices was ensured. No incentives were provided to the respondents. The study was open for 6 weeks between September 30 and November 11, 2016. Reminders were sent to all those who had not replied after 1 and 3 weeks. Due to the characteristics of the survey, a response rate ranging between 15 and 25% was expected.

Data were entered in an Excel database (Microsoft Corporation, Redmond, Washington, USA) and analyzed using Stata 11.0 software package (StataCorp, College Station, TX). Descriptive analyses included medians and interquartile ranges (IQR) for continuous variables or frequency (%) for categorical variables The two-sided chi-square or Fisher’s exact test was used for categorical variables, as appropriate. All tests were two-sided, and *p* values of 0.05 or lower were considered statistically significant.

## Results

### Baseline data: coverage, response rate, working setting, and professional profile

A total of 161 (19.4%) of the 831 experts who were contacted by email completed the survey after two reminders. One incomplete survey was excluded from the study. In two cases the participants were from the same institution and only one survey was considered. One hundred fifty-eight responses were included in our analysis. Participants work settings and professional profiles are summarized in Table 1.

**Table 1:** Participants’ working settings and professional profiles

Characteristics	African region *n* = 8	Eastern- Mediterranean region *n* = 13	European region *n* = 67	Region of Americas *n* = 47	South-East Asia region *n* = 8	Western Pacific region *n* = 15	Total *n* = 158
Type of hospital, n (%)							
- University hospital	5 (62.5)	6 (46.1)	50 (74.6)	35 (74.5)	6 (75.0)	12 (80.0)	114 (72.1)
- Community teaching hospital	2 (25.0)	3 (23.1)	14 (20.9)	9 (19.1)	1 (12.5)	1 (6.7)	30 (19.0)
- Community hospital	0	2 (15.4)	3 (4.5)	1 (2.1)	1 (12.5)	2 (13.3)	9 (5.7)
- Other	1 (12.5)	2 (15.4)	0	2 (4.3)	0	0	5 (3.2)
Hospital setting, n (%)							
- Urban	5 (62.5)	10 (76.9)	65 (97.0)	44 (93.6)	6 (75.0)	14 (93.3)	144 (91.1)
- Suburban	3 (37.5)	3 (23.1)	2 (3.0)	1 (2.1)	2 (25.0)	0	11 (7.0)
- Rural	0	0	0	2 (4.3)	0	1 (6.7)	3 (1.9)
Hospital inpatient beds, n (%)							
- ≤100	0	2 (15.4)	3 (4.5)	1 (2.1)	0	0	6 (3.8)
- 101–500	3 (37.5)	5 (38.5)	15 (22.4)	10 (21.3)	2 (25.0)	3 (20.0)	38 (24.1)
- 501–1000	3 (37.5)	5 (38.5)	27 (40.3)	28 (59.6)	3 (37.5)	1 (6.7)	67 (42.4)
- ≥ 1000	2 (25.0)	1 (7.7)	22 (32.8)	8 (17.0)	3 (37.5)	11 (73.3)	47 (29.7)
Profession, n (%)							
Epidemiologist	1 (12.5)	0	2 (3.0)	1 (2.1)	0	0	4 (2.5)
Hospital administrator	0	0	0	0	0	0	0
Clinical pharmacologist	0	1 (7.7)	0	4 (8.5)	1(12.5)	1 (6.7)	7 (4.4)
Hospital pharmacist	0	0	1 (1.5)	1 (2.1)	1 (12.5)	1 (6.7)	4 (2.5)
Infection control specialist	0	0	1 (1.5)	1 (2.1)	0	0	2 (1.3)
Infectious diseases specialist	0	4 (30.8)	10 (14.9)	10 (21.3)	0	5 (33.3)	29 (18.4)
Intensivist	1 (12.5)	0	5 (7.5)	2 (4.3)	0	1 (6.7)	9 (5.7)
Microbiologist	3 (37.5)	3 (23.1)	1 (1.5)	1 (2.1)	3 (37.5)	0	11 (7.0)
Surgeon	3 (37.5)	5 (38.5)	44 (65.7)	24 (51.1)	3 (37.5)	6 (40.0)	85 (53.8)
Other	0	0	3 (4.5)	3 (6.4)	0	1 (6.7)	7 (4.4)

The response rate was similar to that of previous studies promoted by WSES [1, 7, 8].

As in the other WSES studies [1, 7, 8], participants were not homogeneously distributed across all geographic regions of the world due to the difficulty in recruiting participants in some areas of the world. However all geographic regions were represented in the survey.

### Characteristics of the team

One hundred fifty-six (98.7%) participants stated their hospital had a multidisciplinary AST. Ninety participants (90/156, 57.7%) declared they were currently members of the team, with no difference in frequency between different WHO regions. The median number of physicians working inside the team was five [IQR 4–6]. Characteristics of the team are in Table 2.

**Table 2:** Characteristics of the team in 156 hospitals

Characteristics	*n* (%)
Components	
- Epidemiologist	64 (41.0)
- Hospital administrator	73 (46.8)
- Clinical pharmacologist	8 (5.1)
- Hospital pharmacist	95 (60.9)
- Infection control specialist	106 (67.9)
- Infectious disease specialist	125 (80.1)
- Intensivist	76 (48.7)
- Microbiologist	119 (76.3)
- Surgeon	92 (59.0)
- Other	11 (7.1)
- Infectious disease specialist AND hospital pharmacologist/pharmacist	87 (55.8)
Frequency of meetings	
- More than once a week	15 (9.6)
- Once a week	26 (16.7)
- Twice a month	13 (8.3)
- Once a month	58 (37.2)
- Less than once a month	27 (17.3)
- Only as necessary	17 (10.9)

One hundred thirty-five (135/158, 85.4%) participants had at least one surgeon with an interest or skills in surgical infections within the surgical department of their hospital; a surgeon was significantly more likely to be present in university hospitals (89.5%, two-sided chi-square test *p* < 0.05) compared to community teaching hospitals (83.3%) and community hospitals (66.7%).

### Implementation of protocols and monitoring systems

Implementation of protocols and monitoring systems in 158 hospitals are reported in Table 3.

**Table 3:** Implementation of protocols and monitoring systems in 158 hospitals

Implementation of protocols and monitoring systems	All hospital wards *n* (%)	Some hospital wards, including surgical wards *n* (%)		Some hospital wards, not including surgical wards *n* (%)	No hospital wards *n* (%)
		Every surgical wards	Some surgical wards		
- SAP protocol	NA	124 (78.5)	28 (17.7)	NA	6 (3.8)
- TIS protocol	NA	70 (44.3)	60 (38.0)	NA	28 (17.7)
- UAMS	84 (53.2)	45 (28.5)		9 (5.7)	20 (12.7)
- RDSR	104 (65.8)	26 (16.5)		7 (4.4)	21 (13.3)

*SAP* Surgical antimicrobial prophylaxis, *TIS* therapy for infections in surgery, *UAMS* used antimicrobial monitoring system, *RDSR* resistance data systematic report

The vast majority of respondents (152/158, 96.2%) stated that their hospitals have a protocol for pre-operative prophylaxis. The protocol covered all surgical wards in 124 (78.5%) cases. A protocol for antimicrobial treatment of surgical infections was available in 130 (82.3%) hospitals; however, only 70 (44.3%) had it available in every surgical ward. One hundred twenty-eight (81.0%) hospitals had both a protocol for peri-operative prophylaxis and for antimicrobial treatment of surgical infections available, while four (4/158, 2.5%) hospitals lacked both.

Among 130 surgical wards implementing a protocol for antimicrobial treatment of surgical infections, 97 (74.6%) participants stated it included interventions to reduce the duration of therapy, 88 (67.7%) interventions to switch selected antimicrobials from intravenous-to-oral therapy, 78 (60.0%) interventions for alternative dosing strategies based on pharmacokinetics and pharmacodynamics, with significant difference between community hospitals (11.1%, two-sided Fischer’s exact test *p* < 0.05) compared to university (57.0%) and community teaching (60.0%) hospitals. Thirty-five (26.9%) participants reported the use of biological markers - such as procalcitonin to decrease antimicrobial use in critically ill patients.

### Implementations of ASPs and related interventions

One hundred fifty-five (155/158, 98.1%) participants declared their hospital had an ASP running.

Our survey showed that 30 (19.4%) hospitals have developed persuasive interventions, 17 (11.0%) restrictive interventions and 108 (69.7%) both of them.

Twenty-three surgical departments (23/136, 16.9%) have developed persuasive interventions, 14 (10.3%) restrictive interventions and 99 (72.8%) both of them.

The most common types of interventions in surgical departments were dissemination of educational materials (62.5%), expert approval (61.0%), audit and feedback (55.1%), educational outreach (53.7%), and compulsory order forms (51.5%).

Types of ASPs and related interventions in surgical departments and in all hospital wards are described in detail in Table 4.

**Table 4:** Difference in type of ASPs and related implemented types of interventions in surgical departments and non-surgical departments

Characteristics	Surgical departments, *n* = 136 *n* (%)	Other departments, *n* = 19 *n* (%)	*P* value	Total, *n* = 155 *n* (%)
Type of ASPs				
- Persuasive interventions	23 (16.9)	7 (36.8)	0.06^a^	30 (19.4)
- Restrictive interventions	14 (10.3)	3 (15.8)	0.44^a^	17 (11.0)
- Both	99 (72.8)	9 (47.4)	<0.05	108 (69.7)
Type of interventions				
- Dissemination of educational materials	85 (62.5)	8 (42.1)	0.15	93 (60.0)
- Reminders	56 (41.2)	8 (42.1)	1.00	64 (41.3)
- Audit and feedback	75 (55.1)	4 (21.1)	<0.05	79 (51.0)
- Educational outreach	73 (53.7)	10 (52.6)	1.00	83 (53.6)
- Other persuasive interventions	23 (16.9)	3 (15.8)	1.00^a^	26 (16.8)
- Compulsory order form	70 (51.5)	7 (36.8)	0.34	77 (49.7)
- Expert approval	83 (61.0)	5 (26.3)	<0.05	88 (56.8)
- Restriction by removal	41 (30.1)	2 (10.5)	0.13	43 (27.7)
- Review and make changes	36 (26.5)	1 (5.3)	<0.05^a^	37 (23.9)
- Other restrictive interventions	10 (7.4)	3 (15.8)	0.20^a^	13 (8.4)

All *p* values were calculated using two-sided chi-square test unless otherwise noted

*ASP* antimicrobial stewardship program

^a^Calculated using two-sided Fisher’s exact test

Six (6/41, 14.6%) surgical departments implementing a formulary restriction do not perform any monitoring system of used antimicrobials, and 4 (4/41, 9.8%) do not carry out any systematic reports about resistance data. Furthermore, 6 (7/70, 10.0%) surgical departments using a compulsory order form do not perform any monitoring system of used antimicrobials, and 11 (11/70, 15.7%) do not carry out any systematic reports about resistance data.

One hundred twenty-five (125/158, 79.1%) participants stated their hospital had carried out structural interventions to improve ASPs in the last 5 years. Sixty-nine (43.7%) changed from paper to computerized records, 74 (46.8%) implemented rapid laboratory testing, 32 (20.3%) introduced computerized decision support systems, 69 (43.7%) introduced organization of quality monitoring mechanisms and 29 (18.4%) implemented other structural interventions.

Characteristics of the implementation of protocols, monitoring systems, and ASPs interventions in surgical departments are detailed in Table 5.

**Table 5:** Implementation of protocols, monitoring systems and ASPs interventions in surgical departments related to working setting and team components

Variables	PAP protocol implemented *n*. (%), p	TIS protocol implemented *n*. (%), *p*	Monitoring system of used antimicrobials *n*. (%), p	Resistance data systematic reports *n.* (%), *p*	ASP implemented *n*. (%), *p*	Structural intervention *n*. (%), *p*
Number of bed						
Less than 100, *n* = 6	5 (83.3) 1.00^a^	4 (66.7) 0.29^a^	4 (66.7) 0.30^a^	4 (66.7) 0.29^a^	3 (50.0) 0.58^a^	3 (50.0) 0.10^a^
101–500, *n* = 38	37 (97.4) 1.00^a^	32 (84.2) 0.57	29 (76.3) 0.80	30 (78.9) 1.00	32 (84.2) 0.75	22 (57.9) <0.05^a^
501–1000, *n* = 67	62 (92.5) 0.08^a^	53 (79.1) 0.49	53 (79.1) 0.62	56 (83.6) 0.87	56 (83.6) 0.75	58 (86.6) <0.05^a^
More than 1000, *n* = 47	47 (100.0) 0.18^a^	40 (85.1) 0.71	42 (89.4) 0.16	39 (83.0) 1.00	39 (83.0) 1.00	41 (87.2) 0.16
Hospital setting						
Urban, *n* = 144	140 (97.2) 0.09	118 (81.9) 1.00^a^	119 (82.6) 0.29^a^	122 (84.7) <0.05^a^	120 (83.3) 0.40^a^	97 (67.4) 0.17^a^
Suburban and rural, *n* = 14	12 (85.7) 0.09	12 (85.7) 1.00^a^	11 (78.6) 0.29^a^	9 (64.3) <0.05^a^	10 (71.4) 0.40^a^	9 (64.3) 0.17^a^
Type of hospital						
University hospital, *n* = 114	110 (96.5) 0.67^a^	92 (80.7) 0.55	94 (82.5) 0.84	98 (86.0) 0.08	92 (80.7) 0.84	95 (83.3) <0.05
Community teaching hospital, *n* = 30	29 (96.7) 1.00^a^	27 (90.0) 0.33	27 (90.0) 0.60	24 (80.0) 0.92	26 (86.7) 0.77^a^	22 (73.3) 0.54
Community hospital, *n* = 9	8 (88.9) 0.30^a^	8 (88.9) 1.00^a^	7 (77.8) 0.67^a^	5 (55.6) 0.05^a^	9 (100.0) 1.00^a^	3 (33.3) <0.05^a^
Other, *n* = 5	5 (100.0) 1.00^a^	3 (60.0) 0.21^a^	3 (60.0) <0.05^a^	4 (80.0) 0.21^a^	4 (80.0) 0.52^a^	4 (80.0) 1.00^a^
Components of the team						
Epidemiologist, *n* = 64	62 (96.9) 1.00^a^	53 (82.8) 1.00	52 (81.3) 1.00	59 (92.2) <0.05	52 (81.3) 1.00	53 (82.8) 0.46
Infection control specialist, *n* = 106	103 (97.2) 0.40^a^	90 (84.9) 0.31	91 (85.8) 0.08	89 (84.0) 0.57	89 (84.0) 1.00	82 (77.4) 0.79
Hospital administrator, *n* = 73	71 (97.3) 0.69^a^	65 (89.0) 0.06	61 (83.6) 0.71	61 (83.6) 0.86	63 (86.3) 0.29	59 (80.8) 0.49
Hospital pharmacologist, *n* = 95	94 (98.9) <0.05^a^	80 (84.2) 0.57	80 (84.2) 0.42	82 (86.3) 0.16	83 (87.4) 0.29	79 (83.2) 0.08
Hospital pharmacist, *n* = 8	7 (87.5) 0.27^a^	6 (75.0) 0.43^a^	7 (87.5) 0.55^a^	5 (62.5) 0.15^a^	7 (87.5) 0.36^a^	3 (37.5) <0.05^a^
Infectious diseases specialist, *n* = 125	120 (96.0) 1.00^a^	100 (80.0) v0.23	104 (83.2) 0.47	103 (82.4) 1.00	107 (85.6) 0.15^a^	95 (76.0) 0.50
Intensivist, *n* = 76	74 (97.4) 0.68^a^	65 (85.5) 0.41	66 (86.8) 0.16	63 (82.9) 1.00	66 (86.8) 0.80	60 (78.9) 1.00
Microbiologist, *n* = 119	119 (100.0) 0.64^a^	98 (82.4) 1.00	96 (80.7) 0.75	100 (84.0) 0.44	98 (82.4) 0.82	91 (76.5) 0.46
Surgeon, *n* = 92	88 (95.7) 1.00^a^	80 (87.0) 0.11	77 (83.7) 0.56	77 (83.7) 0.73	76 (82.6) 0.70	70 (76.1) 0.61
Other, *n* = 11	11 (100.0) 1.00^a^	9 (81.8) 1.00^a^	11 (100.0) 0.22^a^	10 (90.9) 0.69^a^	10 (90.9) 1.00^a^	8 (72.7) 0.70^a^

All *p* values were calculated using two-sided chi-square test unless otherwise noted

*PAP* pre-operative antimicrobial prophylaxis, *TIS* therapy for infections in surgery, *ASP* antimicrobial stewardship program

^a^Calculated using two-sided Fisher’s exact test. *ASP* antimicrobial stewardship program

## Discussion

Antimicrobial stewardship programs (ASP) are a key strategy to curb the spread of antibiotic resistance [3, 9]. The best strategies for an ASP are not definitively established and are likely to vary based on local routine clinical practice [7], despite several guidelines on the topic [9, 10].

Successful ASPs should focus on collaboration between healthcare professionals in order to share knowledge and best practices. It is essential for an ASP to have at least one member who is an infectious diseases specialist. Pharmacists with advanced training or longstanding clinical experience in infectious diseases are also key actors for the design and implementation of the stewardship program interventions [11]. Infection control specialists and hospital epidemiologists should coordinate efforts on monitoring and preventing healthcare-associated infections and in analyzing and reporting “real-time” data to prevent infections, improve antimicrobial use, and minimize secondary spread of resistance. Microbiologists should actively guide the proper use of tests and the flow of laboratory results. Being involved in providing surveillance data on antimicrobial resistance, they should provide periodic reports on antimicrobial resistance data allowing the multidisciplinary team to determine the ongoing burden of antimicrobial resistance in the hospital. Moreover, timely and accurate reporting of microbiology susceptibility test results allows selection of more appropriate targeted therapy, and may help reduce broad-spectrum antimicrobial use.

Surgeons with adequate knowledge in surgical infections and surgical anatomy when involved in ASPs may audit antibiotic prescriptions, provide feedback to the prescribers and integrate best practices of antimicrobial use among surgeons, and act as champions among colleagues. Although many surgeons are aware of the problem of antimicrobial resistance, most underestimate it in their own hospital [1]. Very few studies have been published on the role of ASPs in general surgical departments. In 2015, Cakmakci [12] suggested that the engagement of surgeons in ASPs might be crucial to their success. In 2013, however, Duane et al. showed poor compliance of surgical services with ASP recommendations [13]. Surgeons need to take part in addressing the global issue of antimicrobial resistance. Failure to do so will be catastrophic to patients and programs [3].

Infections are the main factors contributing to mortality in intensive care units (ICU) [14].

Intensivists have a critical role in treating multidrug resistant organisms in ICUs in critically ill patients. They have a crucial role in prescribing antimicrobial agents for our most challenging patients and are at the forefront of a successful ASP [15].

Finally, without adequate support from hospital administration, the ASP will be inadequate or inconsistent since the programs do not generate revenue [16]. Engagement of hospital administration has been confirmed as a key factor for both developing and sustaining an ASP [17].

In most cases, our survey demonstrated that ASPs do not involve a true multi-disciplinary approach.

An infectious diseases specialist and a hospital pharmacist were part of the team in 125 (80.1%) and in 95 (60.9%) cases, respectively. Only 87 (55.8%) teams included both an infectious diseases specialist and a hospital pharmacist. An infection control specialist and a hospital epidemiologist were part of the team in 106 (67.9%) and in 64 (41.0%) cases, respectively. It is possible that in some hospitals, AMS and infection prevention and control team are two separate entities, which collaborate. A microbiologist was part of the team in 119 (76.3%) cases. A surgeon was part of the team in 92 (59.0%) cases and an intensivist in 76 (48.6%) cases. A hospital administrator was part of the team only in 73 (46.8%) cases. Interestingly a surgeon was significantly more likely to be part of the team in university hospitals (89.5%, two-sided chi-square test *p* < 0.05) compared to community teaching (83.3%) and community non-teaching hospital (66.7%).

Strategies of ASPs should be tailored based on individual hospital characteristics and personnel and resources available. The Infectious Diseases Society of America/Society for Healthcare Epidemiology of America (IDSA/SHEA) guidelines identified two core proactive evidence-based strategies and several supplemental strategies for promoting antimicrobial stewardship [7, 8]: first, a restrictive strategy based on a proactive strategy of either formulary restriction or a requirement for pre-approval for specific drugs or both, and second, a persuasive strategy of performing prospective audit with intervention and feedback to the prescriber.

Our survey showed that 23 (16.9%) surgical departments have developed persuasive interventions, 14 (10.3%) restrictive interventions and 99 (72.8%) both of them. ASP policies should be based on both international/national antibiotic guidelines, and tailored to local microbiology and resistance patterns. Local clinical practice guidelines and algorithms can be an effective way to standardize prescribing practices based on the country’s epidemiology. Standardizing a shared protocol of antimicrobial prophylaxis should represent the first step of any Antimicrobial Stewardship program.

One hundred fifty-two (96.2%) participants stated their hospitals have a protocol for surgical antibiotic prophylaxis. Among the 158 hospitals, a protocol for antibiotic prophylaxis is present in all surgical wards in 124 (78.5%) of hospitals while only in some surgical wards in 28 (17.7%) hospitals.

A protocol for antibiotic treatment was present in all surgical wards in 70 (44.3%) hospitals, while only in some surgical wards in 60 (38.0%) hospitals. Among 130 hospitals implementing a protocol for antimicrobial treatment of surgical infections, 97 (74.6%) participants stated that it included interventions to reduce the duration of therapy, 88 (67.7%) interventions to switch select antimicrobials from intravenous-to-oral therapy, 78 (60.0%) interventions for alternative dosing strategies based on pharmacokinetic and pharmacodynamic principles, with substantial difference between community hospitals (11.1%, two-sided Fischer’s exact test *p* < 0.05), university (57.0%) and community teaching (60.0%) ones. Thirty-five (26.9%) participants admitted to the use of biological markers - such as procalcitonin - to decrease antimicrobial use in critically ill patients**.**

In any healthcare setting, a significant amount of time and energy should be spent on infection control. Surveillance studies can help clinicians to identify trends in pathogens incidence and antimicrobial resistance, including identification of emerging pathogens at local level*.* The survey showed that 130 (83.3%) surgical departments had systematic reports about resistance data.

Hospital pharmacists inside the multidisciplinary team should negotiate with hospital administration to obtain adequate and necessary infrastructure to measure antimicrobial use. Regular feedback about antimicrobial consumption can be an important determinant for change for healthcare professionals and policy makers to expedite progress towards prudent use of antimicrobials. The survey showed that 129 (81.6%) surgical departments had an antimicrobial monitoring system.

Interestingly, 6 (6/41, 14.6%) surgical departments implementing a formulary restriction do not perform any monitoring system of used antimicrobials, and 4 (4/41, 9.8%) do not carry out any systematic reports about resistance data. Furthermore, 6 (7/70, 10.0%) surgical departments using a compulsory order form do not perform any monitoring system of used antimicrobials, and 11 (11/70, 15.7%) do not carry out any systematic reports about resistance data. In institutions that use restrictive interventions, monitoring overall trends in antimicrobial use and systematic reports about resistance data should be necessary to assess and respond to such shifts in use.

The ultimate goal of any stewardship program should be to stimulate a behavioral change in prescribing practices. In this context, education of prescribers is crucial to convince clinicians to use antibiotics judiciously. However, without concurrent interventions education alone is of little value. In this regard, various stewardship interventions have been implemented with the aim of improving adherence to guidelines. Where these interventions have been clinician focused, accumulating evidence suggests that educational interventions are mostly ineffective and result in insignificant changes to overall compliance [17]. It is possible that this might relate to cognitive dissonance, a process in which clinician-focused education fails to engage prescribers effectively, allowing them to ignore the evidence and to continue with their regular habits and practices. Alternative strategies of improving antibiotic management of surgical patients are needed and these may include guidance of clinicians in the institutional process of improvement, which has not as yet been addressed in guidelines [17]. The answer may lie within the principles and imperatives contained with the change of processes in hospitals.

It is highly important that faculty in academic medical centers and teaching hospitals focus on fundamental antibiotic stewardship principles in their preclinical and clinical curricula [18].

The survey found that dissemination of educational materials and educational outreach were developed respectively in 85 cases (62.5%) and 73 (53.7%) surgical departments.

This study has several limitations: with a response rate of just 19.4% we have to consider a response bias, and it is possible that non-participating physicians may have been less interested in ASPs than the participants and therefore it is possible that results are biased towards a better picture than it actually is. Furthermore, the study was conducted in a sample of physicians who participated in the AGORA project, and selecting international experts in the field again potentially resulting in an overrepresentation of hospitals with a considerably active ASP. No stratification or sampling according to medical specialty were pre-planned to ensure that all stakeholders were adequately represented, and finally our questionnaire was self-reported, has not been externally validated, and was evaluated in a single institution. The major strength of the study is its multinational (global) and multidisciplinary approach, to our best knowledge the first in this setting. Thus, our survey provides a benchmark to all interested stakeholders; it can be repeated over time to explore if better uniformity on a global platform of healthcare environments would develop in the future, and may be used to build consensus around the best practices in the field of prevention of surgical infections and rational use of antibiotics in a future project.

## Conclusions

The results of the survey showed a heterogeneous organization of ASPs worldwide and demonstrated the need for a cohesive approach in order limit the emergence of antimicrobial resistance in surgical infections. Successful ASPs should focus on collaboration between all healthcare professionals in order to gain the wider-possible acceptance, share knowledge and spread best clinical practices. The main bias of the survey is the low response rate.

## Additional file

Additional file 1: The international cross-sectional survey. (DOC 56 kb)

## Abbreviation

ASP: Antimicrobial stewardship program
