# Supplementary material for: The Global Alliance for Infections in Surgery: defining a model for antimicrobial stewardship—results from an international cross-sectional survey
Source: World J Emerg Surg. 2017 Aug 1;12:34. doi: 10.1186/s13017-017-0145-2 (PMC5540347; doi:10.1186/s13017-017-0145-2)
Supplement: Additional file 1: — The international cross-sectional survey. (DOC 56 kb) [file 13017_2017_145_MOESM1_ESM.doc]

**Additional file 1**

*Working setting*

**1. What country are you working in?**

**2. What kind of hospital are you working in?**

University hospital _1_

Community hospital _2_

Community teaching hospital _3_

Other _4_

**3. What is the setting of your hospital?**

Urban _1_

Suburban _2_

Rural _3_

**4. How many inpatient beds are placed in your hospital?**

Less than 100 _1_

101-500 _2_

251-500 _3_

501-1000 _4_

More than 1000 _5_

*Professional profile*

**5. What is your profession?**

Epidemiologist _1_

Hospital administrator _2_

Hospital Pharmacologist _3_

Infection Control Specialist _4_

Infectious Diseases Specialist _5_

Intensivist _6_

Microbiologist _7_

Surgeon _8_

Other _9_

**6. Does your hospital have a multidisciplinary antimicrobial stewardship and infection control team?**

Yes No

1 2

**7. Are you currently a member of the team?**

Yes No

1 2

*Characteristics of the surgical infection prevention and control team*

**8. Which physicians does the team include?**

Epidemiologist _1_

Hospital administrator _2_

Hospital Pharmacologist _3_

Infection Control Specialist _4_

Infectious Diseases Specialist _5_

Intensivist _6_

Microbiologist _7_

Surgeon _8_

Other _9_

**9. How many times does the team meet?**

More than once a week _1_

Once a week _2_

Twice a month _3_

Once a month _4_

Less than once a month _5_

Only as necessary _6_

**10. Is there at least a surgeon with interest/skills in surgical infections inside the surgical department of your hospital?** Yes No

1 2

*Existence of protocols*

**11. Does your hospital have a protocol for pre-operative antimicrobial prophylaxis?**

Yes, all the surgical wards have got one _1_

Yes, but only some surgical wards have got one _2_

No _3_

**12. Does your hospital have a protocol for antimicrobial treatment of surgical infections?**

Yes, all the surgical wards have got one _1_

Yes, but only some surgical wards have got one _2_

No _3_

**13. Does the protocol for antimicrobial treatment of surgical infections consider interventions to reduce the duration of therapy?**

Yes No

1 2

**14. Does the protocol for antimicrobial treatment of surgical infections recommend interventions to switch select antimicrobials from intravenous-to-oral therapy?**

Yes No

1 2

**15. Does the protocol for antimicrobial treatment of surgical infections advocate for alternative dosing strategies based on pharmacokinetic/pharmacodynamic principles?**

Yes No

1 2

**16. Does the protocol for antimicrobial treatment of surgical infections advocate biological markers - such as Procalcitonin testing - as an intervention to decrease antimicrobial use in critically ill patients?**

Yes No

1 2

*Existence monitoring systems*

**17. Does your hospital perform a monitoring system of used antimicrobials?**

Yes, all the surgical wards have got one _1_

Yes, but only some hospital wards are monitored, including surgical wards _2_

Yes, but only some hospital wards are monitored, not including surgical wards _3_

No _4_

**18. Does your hospital periodically carry out systematic reports about resistance data?**

Yes, all the surgical wards have got one _1_

Yes, but only some hospital wards are monitored, including surgical wards _2_

Yes, but only some hospital wards are monitored, not including surgical wards _3_

No _4_

*Features of the ASP and related performed interventions*

**19. Does your hospital periodically carry out systematic reports about resistance data?**

Yes, all the surgical wards have got one _1_

Yes, but only some hospital wards are monitored, including surgical wards _2_

Yes, but only some hospital wards are monitored, not including surgical wards _3_

No _4_

**20. Which kind of interventions is the ASP based on?**

Persuasive interventions _1_

Restrictive interventions _2_

Both of them _3_

**21. What kind of persuasive interventions are in place?**

Dissemination of educational materials in printed form or via educational meetings _1_

Reminders _2_

Audit and feedback _3_

Educational outreach _4_

Other persuasive interventions _5_

No one _6_

**22. What kind of restrictive interventions are in place?**

Compulsory order form _1_

Expert approval _2_

Restriction by removal _3_

Review and make change _4_

Other restrictive interventions _5_

No one _6_

**23. Did your hospital carry out structural interventions finalized to improve ASPs in the last 5 years?**

Yes No

1 2

**24. What kind of structural interventions have been carried out?**

Changing from paper to computerized records _1_

Rapid laboratory testing _2_

Computerized decision support system _3_

Organization of quality monitoring mechanisms _4_

Other structural interventions _5_

No one _6_
